# Inhibition of Quorum Sensing-Controlled Virulence Factors and Biofilm in *Pseudomonas aeruginosa* by *Piper* Species

**DOI:** 10.3390/antibiotics15060627

**Published:** 2026-06-22

**Authors:** Juliet A. Prieto-Rodriguez, Lida V. Hernández-Moreno, Ludy C. Pabón-Baquero, Oscar J. Patiño-Ladino, Luis E. Cuca-Suárez

**Affiliations:** 1 Departamento de Química, Facultad de Ciencias, Pontificia Universidad Javeriana, Bogotá 110231, Colombia; 2 Departamento de Química, Facultad de Ciencias, Universidad Nacional de Colombia, Sede Bogotá, Bogotá 111321, Colombia; lhernandezmo@unal.edu.co (L.V.H.-M.); ojpatinol@unal.edu.co (O.J.P.-L.); lecucas@unal.edu.co (L.E.C.-S.); 3 Escuela de Ciencias Básicas y Aplicadas, Universidad de la Salle, Bogotá 111711, Colombia; lupabon@unisalle.edu.co

**Keywords:** Piperaceae, *Piper* spp., quorum sensing, *P. aeruginosa*, antimicrobial resistance, biofilm, virulence factors

## Abstract

**Background:** The World Health Organization has identified the growing ineffectiveness of antibiotics against resistant pathogens as a global threat to public health, linked to increased morbidity and mortality. In this context, *Pseudomonas aeruginosa* stands out as a multidrug-resistant, biofilm-forming pathogen whose biofilm formation increases its tolerance to antimicrobials, which has driven the development of anti-virulence strategies as a therapeutic alternative. In this regard, the present study aimed to evaluate extracts and compounds from *Piper* species in assays targeting the inhibition of biofilm and virulence factors in *Pseudomonas aeruginosa*, as well as their anti-quorum sensing activity using *Chromobacterium violaceum* as a biosensor model. **Methods:** For this purpose, quorum sensing interference was first assessed through inhibition of violacein production using *C. violaceum* ATCC 12472 as a biosensor model. The modulation of virulence-associated phenotypes in *P. aeruginosa* ATCC BAA-47 was subsequently examined through inhibition of biofilm formation by crystal violet staining and spectrophotometric quantification of elastase, protease and pyocyanin production. **Results:** It was found that extracts from *P. aduncum*, *P. sucrense*, *P. grande*, and *P. cumanense* inhibited biofilm formation in *P. aeruginosa* and showed potential activity against quorum sensing in the *C. violaceum* model, while *P. ceanothifolium* exhibited only antibiofilm activity. Furthermore, hydroquinone-type compounds and benzoic acid derivatives reduced biofilm formation and virulence factors in *P. aeruginosa*. **Conclusions:** The results obtained demonstrate antibiofilm and anti-virulence activity, as well as a possible modulation of quorum sensing in model systems, suggesting that *Piper* species represent a promising source of bioactive compounds.

## 1. Introduction

*Pseudomonas aeruginosa* is a Gram-negative opportunistic pathogen frequently associated with nosocomial and chronic infections, particularly in immuno-compromised patients. Although not all clinical isolates are multidrug-resistant (MDR), the increasing prevalence of MDR strains has markedly complicated treatment and infection control [1,2,3,4]. The clinical challenge posed by MDR *P. aeruginosa* is particularly evident in clinical infections, where surveillance studies have documented alarmingly high rates of resistance to multiple antibiotic classes, including β-lactams [5]. Recent studies highlighted a direct mechanistic link between biofilm-forming capacity and both virulence factor expression and antibiotic resistance in *P. aeruginosa* [6]. The inclusion of biofilm formation, virulence, and multidrug resistance emphasizes the urgent need for alternative therapeutic strategies, which can disrupt pathogenic mechanisms. Its persistence is driven by the interplay of multiple resistance mechanisms, including low outer-membrane permeability, efflux-mediated extrusion, antibiotic-inactivating enzymes, and acquired resistance determinants, together with a marked ability to form biofilms. This biofilm lifestyle further enhances tolerance to antimicrobial treatment, while the production of virulence factors such as elastase, pyocyanin, alkaline proteases, and rhamnolipids contributes to tissue damage, immune evasion, and chronic infection [7,8,9]. Because many of these pathogenic traits are coordinately regulated via quorum sensing (QS), this system has emerged as an attractive target for alternative anti-infective strategies [10,11,12].

In Gram-negative bacteria, QS commonly depends on N-acyl-homoserine lactones (AHLs) that bind to LuxR-type receptors; in *P. aeruginosa*, this regulatory framework is mainly represented by the Las and Rhl systems and is functionally interconnected with the alkyl-quinolone-based Pqs circuit. Together, these systems coordinate the expression of key traits linked to pathogenicity and persistence, including the production of pyocyanin, elastases, proteases, and rhamnolipids, as well as biofilm development and maturation [13,14,15]. Because QS regulates virulence-associated phenotypes rather than bacterial growth itself, the search for quorum-sensing inhibitors (QSIs) has gained considerable interest as a potential therapeutic strategy against *P. aeruginosa*, with the added advantage of potentially reducing the selective pressure commonly associated with conventional bactericidal or bacteriostatic treatments [11,12,13,14,15]. QS-mediated communication can be disrupted at different levels, including inhibition of autoinducer synthesis, enzymatic degradation of signaling molecules, or competition for binding to receptor proteins, ultimately blocking target gene expression through interfering molecules known as QSIs. Current efforts to identify such compounds have relied on experimental screening and structure–activity relationship-guided approaches, which have enabled the discovery of multiple active scaffolds; however, these strategies still face important limitations, including dependence on finite experimental datasets, high costs, labor-intensive workflows, relatively low throughput, and often limited mechanistic insight, highlighting the need to continue exploring new sources of chemically diverse QSI candidates [11,12,13,14,15,16].

Plants have been extensively explored as sources of QSIs, and both crude extracts and specialized metabolites have been shown to modulate QS-regulated phenotypes in *P. aeruginosa*, including biofilm formation, motility, and virulence-factor production, at sub-inhibitory concentrations [17,18,19]. In this context, the genus *Piper* in the family Piperaceae is notable for its rich chemical diversity, encompassing amides, phenylpropanoids, benzoic acid derivatives, terpenoids, flavonoids, and other metabolite classes associated with antimicrobial and antivirulence activities [20,21,22,23,24]. Accordingly, extracts from species such as *P. betle* L., *P. nigrum* L. and *P. longum* L. have been reported to interfere with QS-related phenotypes in Gram-negative bacteria, including violacein production, virulence-associated traits, and biofilm [25,26,27]. At the molecular level, chavicol acetate and acetoxychavicol acetate from *Piper betle* have demonstrated in silico binding affinity for CviR and LasR proteins, respectively, while a dihydroxylated derivative of piperlongumine, present in other species of the genus, has demonstrated anti-QS activity through experimental evaluation, highlighting the importance of *Piper* as a source of QS-modulating compounds [28,29].

Although the genus *Piper* is highly diverse in Colombia, with approximately 400 recorded species [30,31,32], only a small proportion has been investigated for quorum-sensing (QS) inhibitory activity. Previous studies showed that the essential oils of *P. bredemeyeri* J.Jacq, *P. brachypodon* (Benth) C.DC. and *P. bogotense* C.DC. inhibited violacein production in *Chromobacterium violaceum,* providing preliminary evidence of anti-QS effects, although *P. aeruginosa* was not directly evaluated [25]. More recently, our research group demonstrated the anti-QS and antibiofilm activity of extracts from *P. bogotense* and *P. pertomentellum* Trel. & Yunck and showed that some of their constituents were able to attenuate virulence-associated factors and biofilm formation in *P. aeruginosa* [33,34].

In this context, the aim of this study was to use *C. violaceum* as a biosensor model to assess the quorum-sensing inhibitory activity of *Piper* species extracts, while also characterizing the ability of these extracts and their chemical constituents to reduce biofilm formation and virulence-associated phenotypes in *P. aeruginosa*.

## 2. Results and Discussion

### 2.1. Effect of Piper Extracts on the Growth of P. aeruginosa

The effect of the 18 ethanolic extracts on the growth of *P. aeruginosa* was evaluated at concentrations of 1000, 250 and 62.5 μg/mL. At the highest concentration (1000 μg/mL), eight extracts produced statistically significant reductions in bacterial growth, *P. aduncum* L. (inflorescences), *P. ceanothifolium* Kunth (inflorescences), *P. cumanense* Kunth (aerial part), *P. falcifolium* Trel. (leaves), *P. nigrum* (seeds), *P. peltatum* L. (inflorescences), *P. statarium* Trel. & Yunk (aerial part) and *P. umbellatum* L. (leaves), the latter two showing the greatest inhibitory effect. At 250 μg/mL, only *P. peltatum* (inflorescences) maintained a significant reduction. At 62.5 μg/mL, no extract significantly affected bacterial growth compared to the untreated control (*p* > 0.05), so this concentration was selected as the working concentration for subsequent QS inhibition and biofilm formation assays (Table S1 in Supplementary Materials). The absence of growth inhibition at 62.5 μg/mL in all extracts is consistent with the methodological premise underpinning anti-QS studies: the use of sub-inhibitory concentrations ensures that the observed reductions in virulence phenotypes reflect a direct interference with QS regulatory circuits and not an artifact derived from the reduction in bacterial biomass [35,36,37]. This approach is articulated with the antivirulence principle of QS inhibition, which seeks to attenuate bacterial pathogenicity without exerting lethal selective pressure on the bacterium [38]. The results obtained for the species with previous reports are mostly consistent with the available literature. For *P. nigrum* (seeds), significant inhibition only at 1000 μg/mL is consistent with the high documented minimum inhibitory concentration (MIC) values for crude extracts of this species against *P. aeruginosa* [39]. For *P. eriopodon* Miq. (aerial part), the absence of activity is consistent with what was reported by Vallejo et al. [40] with dichloromethane extract at the same concentration, suggesting that this species lacks direct antimicrobial activity against this pathogen regardless of the solvent used. The activity of *P. umbellatum* (leaves) at 1000 μg/mL also coincides with the report of Vallejo et al. [40], confirming the reproducibility of the effect between different types of extract. In the case of *P. pesaresanum* C.DC. (Areal part), the activity reported with dichloromethane extract by Vallejo et al. [40] is not reproduced with the ethanolic extract evaluated in this study, a difference that can be attributed to the different phytochemical profiles associated with the polarity of the extraction solvent. Regarding *P. aduncum*, Kloucek et al. [41] reported no inhibitory activity of its ethanolic extract against *P. aeruginosa* ATCC 27853 (MIC >16 mg/mL), while Purayil et al. [42] documented antibacterial activity of the chloroform extract against an MDR clinical isolate of *P. aeruginosa* with a MIC of 1.25 mg/mL, a value considerably higher than the concentrations employed in the present study. For the remaining species with activity at 1000 μg/mL, *P. ceanothifolium*, *P. cumanense*, *P. falcifolium*, *P. peltatum* and *P. statarium*, no previous reports were found against *P. aeruginosa*, so these results constitute the first antecedents of antimicrobial activity of these species against this pathogen.

### 2.2. Inhibition of Violacein Production in C. violaceum and Biofilm Formation in P. aeruginosa by Ethanolic Extracts of Piper Species

The activity of the extracts was further examined using two phenotypic assays: inhibition of violacein production in *C. violaceum* as a biosensor-based indicator of quorum-sensing interference, and inhibition of biofilm formation in *P. aeruginosa* (Table 1). In the biofilm assay, 8 of the 18 extracts significantly reduced biofilm formation. The strongest effects were observed in *P. aduncum* (leaves), *P. aduncum* (inflorescences), *P. aduncum* (stems) and *P. ceanothifolium* (inflorescences), which reduced biofilm formation to 49.2%, 45.9%, 39.2%, and 39.1% of the untreated control, respectively. In the *C. violaceum* assay, 13 extracts significantly reduced violacein production at 62.5 µg/mL, with *P. aduncum* (leaves), *P. grande* (leaves), *P. haughtii* (leaves), *P. nigrum* (seeds) and *P. pesaresanum* (aerial part) reducing pigment production to 50% of the control or less.

The phenotypic profile of *P. aduncum* (leaves) was the most consistent across both assays, reducing violacein production in *C. violaceum* to 45.7% of the control and biofilm formation in *P. aeruginosa* to 49.2%, suggesting that its chemical constituents may interfere with signaling pathways relevant to both biosensor and pathogen models. Similarly, extracts of *P. cumanense* (aerial part), *P. grande* (leaves) and *P. sucrense* (leaves) caused significant inhibition in both assays, indicating activity compatible with modulation of QS-associated phenotypes in both microorganisms. Clinical observations suggest that inhibiting biofilm formation with sub-MIC is significant, given that biofilms produced by strains of *P. aeruginosa* show increased virulence and multidrug resistance [43]. Plant-derived compounds disrupting biofilms may restore antibiotic susceptibility, offering promising adjunctive therapeutic strategies [44,45]. However, the specific mechanisms underlying these effects cannot be established from phenotypic data alone. It should be noted that violacein inhibition in *C. violaceum* reflects interference with an AHL-based QS circuit in a biosensor model and does not constitute direct evidence of QS inhibition in *P. aeruginosa*, whose regulatory network is considerably more complex, encompassing at least three interconnected QS systems (Las, Rhl, and Pqs) that operate hierarchically and regulate overlapping sets of virulence genes [46,47]. For those extracts that reduced biofilm formation without significantly affecting violacein production, *P. aduncum* (inflorescences), *P. ceanothifolium* (inflorescences) and *P. umbellatum* (leaves), the underlying mechanisms may be independent of AHL-mediated QS pathways, and alternative regulatory systems such as c-di-GMP second messenger signaling or the sensor kinase networks GacS/GacA and RetS/LadS, which govern the planktonic-to-biofilm transition in *P. aeruginosa*, could represent plausible mechanistic frameworks, though further experimental evidence would be required to evaluate these possibilities [38,48].

It should be noted that quercetin was used as positive control at 3.9 µg/mL, a concentration approximately 16-fold lower than the working concentration of the extracts (62.5 µg/mL). This concentration was selected based on its previously reported biofilm inhibitory concentration (BIC) for *P. aeruginosa* [33]. Accordingly, a direct quantitative comparison between the positive control and the extracts is not appropriate; quercetin was included to validate assay performance and sensitivity rather than to serve as a potency benchmark.

To the best of our knowledge, this study provides the first report of anti-QS and antibiofilm evaluation against *P. aeruginosa* for the majority of the *Piper* species examined, including *P. annulatispicum* Trel. & Yunck, *P. ceanothifolium*, *P. cumanense*, *P. cundinamarcanum* Trel., *P. eriopodon*, *P. falcifolium*, *P. grande* Vahl., *P. haughtii* Trel & Yunck, *P. peltatum*, *P. pesaresanum*, *P. statarium*, *P. sucrense* Trel. & Yunck., and *P. umbellatum*. This gap in the literature underscores the relevance of systematic screening approaches for understudied botanical genera with documented bioactive potential.

For species with previous reports, the ethanolic extract of *P. nigrum* at 500 µg/mL is reported to inhibit violacein production in *C. violaceum* by 40%, exhibiting an effect similarly to that observed for the methanolic extract (78%) and hexane, chloroform, and methanol fractions, though with no significant activity against *P. aeruginosa* biofilms. It has been described in the literature that the ethanolic extract of *P. bogotense* at a 1000 µg/mL concentration produced only 5% violacein in *C. violaceum* and at 62.5 µg/mL formed 19.8% of biofilm in *P. aeruginosa* [34]. It is also reported for the ethanolic extract of *P. pertomentellum* that it can reduce violacein production with an IC_50_ of 149.6 µg/mL and biofilm formation with a BIC of 3.9 µg/mL [33]. With respect to biofilm inhibition, Purayil et al. [42] reported that the chloroform extract of *P. aduncum* reduced *P. aeruginosa* biofilm formation in a concentration-dependent manner at concentrations ranging from 4 to 16 mg/mL, which are substantially higher than those used in the present work.

### 2.3. Effect of Piper Compounds on the Growth of P. aeruginosa

The concentration range of 250–62.5 µg/mL was selected based on sub-inhibitory concentrations previously reported for plant-derived phytochemicals with anti-quorum sensing activity against *P. aeruginosa* and was consistent with the concentration used for the cinnamic acid positive control [38,48,49]. Prior to the evaluation of biofilm formation and virulence factor production, the effect of the 13 *Piper*-derived compounds on the growth of *P. aeruginosa* was assessed at the concentrations used in the subsequent assays (250–62.5 µg/mL). None of the compounds significantly inhibited bacterial growth at any of the concentrations evaluated, confirming that the working concentrations were sub-inhibitory and that any observed reductions in QS-associated phenotypes can be attributed to direct interference with virulence-related regulatory pathways rather than to a decrease in bacterial biomass [35,36,37]. For clarity and to facilitate comparison across the 21 compounds, Table 2 reports, for each compound and phenotype, the optimal concentration within the tested range, defined as the concentration at which the lowest biofilm formation and the lowest virulence factor production were observed relative to the untreated control (i.e., the concentration yielding the greatest inhibitory effect). The corresponding percentage of reduction is shown in parentheses.

### 2.4. Potential of Compounds from Piper Species in Biofilm Formation and Virulence Factor Production in P. aeruginosa

The isolated compounds of some species of the genus *Piper* were evaluated for their ability to modulate biofilm formation and the production of protease, elastase, and pyocyanin in *P. aeruginosa* (Table 2).

In the biofilm assay, four compounds, **C6**, **C7**, **C10** and **C13**, reduced biofilm formation to below 50% of the untreated control at concentrations below 200 µM. Among them, **C10** and **C13** showed the most pronounced effects, reducing biofilm formation to 43.9% and 44.5% of the control, respectively, whereas **C6** was the most active compound on a molar basis in this essay. Although none of the evaluated compounds outperformed quercetin, these results remain relevant because they reveal biofilm inhibition for several *Piper*-derived metabolites for which such activity had not previously been reported.

Piperine (**C1**) has previously been reported to lack biofilm inhibitory activity [39], which is consistent with the findings of this work. The remaining active compounds belong to chemical classes of amides, hydroquinones, and benzoic acid derivatives for which antibiofilm activity against *P. aeruginosa* has been documented, providing a relevant framework for interpreting the results obtained here. Among the amides, benzamide and cefaradione B, isolated from *P. pertomentellum*, reduced biofilm formation at 34.2% and 41.5% of the untreated control to 31.2 μg/mL, respectively [33]. The sesquiterpene hydroquinone avarol has been shown to reduce *P. aeruginosa* biofilm formation by 75% [50], while the synthetic amide N-cyclopentyl-5-(3-nitrophenyl)-5-oxo-pentamide, which acts on PqsR/LasR signaling, reduced biofilm formation by 45% at 10 μM [51]. Among benzoic acid derivatives, vanillic acid reduced *P. aeruginosa* biofilm formation by 46% at 4 mmol/L [52]. In this context, these precedents support the biofilm inhibitory potential observed for the structurally related compounds identified in this study, although direct potency comparisons are limited by the differences in experimental conditions and concentration units reported in the different studies.

Compounds **C1**–**C3**, **C8** and **C10**–**C12** reduced protease production in *P. aeruginosa*, representing the first report of this activity for all of them. The lowest production percentages were recorded for **C1** (57.2 ± 2.6%; 125 µg/mL), **C8** (57.3 ± 17.1%; 250 µg/mL) and **C10** (49.7 ± 10.6%; 31.2 µg/mL). When comparing **C1** and isopiperine (**C3**), both evaluated at the same concentration (125 µg/mL), **C1** showed greater inhibition (57.2% vs. 74.5% production), which may suggest a preliminary trend in which the geometric configuration of the double bond influences activity; however, this observation should be interpreted cautiously given the limited number of compounds compared. Similarly, since **C1** and **C2** were evaluated at different concentrations, no valid conclusions can be drawn regarding the effect of nitrogen substitution on the amide group, and this comparison is therefore not discussed further. Within the genus *Piper*, hydroquinones and amides from other species have previously shown inhibitory activity against protease production in *P. aeruginosa* ATCC BAA-47. 2-Farnesylhydroquinone from *P. bogotense* reduced production to 73% at 250 µg/mL [34], while the amides ethyltembamide and cepharadione B from *P. pertomentellum* reduced it to 76.7% (125 µg/mL) and 77.1% (250 µg/mL), respectively [33]. The results obtained herein are comparable to those previously reported.

Compounds **C1**–**C3**, **C6**, **C7**, **C10**, **C11** and **C13** reduced elastase production by more than 60% relative to the positive control (cinnamic acid, 42.6 ± 6.4%; 250 µg/mL). The lowest production percentages were recorded for **C1** (43.3 ± 9.6%; 125 µg/mL), **C10** (25.9 ± 14.4%; 31.2 µg/mL) and **C13** (47.2 ± 12.5%; 31.2 µg/mL). When comparing **C10** and **C13**, both evaluated at the same concentration (31.2 µg/mL), **C10,** which bears a longer prenylated chain, showed more pronounced inhibition than **C13**. This difference may suggest that alkyl chain length has some influence on elastase inhibitory activity; however, with only two compounds available for comparison, this observation constitutes a preliminary trend that requires further experimental confirmation. No comparisons are made between compounds tested at different concentrations. This is the first report of elastase inhibitory activity for all compounds evaluated (**C1**–**C13**). Within the genus *Piper*, 3-farnesyl-4-hydroxybenzoic acid and 2-farnesylhydroquinone from *P. bogotense* reduced elastase production to 60% and 51%, respectively, at 250 µg/mL [34], while the amides ethyltembamide and cepharadione B from *P. pertomentellum* reduced production to 48.8% (125 µg/mL) and 57.1% (62.5 µg/mL), respectively [33]. The results of the present work expand the repertoire of *Piper* metabolites with activity against this virulence factor.

Compounds **C1**, **C2**, **C6**–**C8**, **C10**, **C11** and **C13** reduced pyocyanin production to at least 60% of the untreated control. **C10** exhibited the most pronounced inhibitory effect, with a production percentage of 32.6 ± 2.5% at 31.2 µg/mL (120 µM), followed by **C6** (22.7 ± 4.5%; 15.6 µg/mL) and **C1** (27.6 ± 5.3%; 125 µg/mL). When comparing **C7** and **C8**, both benzoic acid derivatives evaluated at different concentrations (31.2 and 250 µg/mL, respectively), differences in inhibitory activity are observed; however, since the concentrations are not equivalent, these differences cannot be attributed to specific structural features such as the presence of a methoxy or hydroxyl group. This comparison is noted as a descriptive observation that may guide future studies using equivalent concentrations. A prior report exists for **C1** (piperine) describing pyocyanin inhibition at concentrations below 50 µg/mL [39], whereas in this work activity was evaluated at 125 µg/mL, which limits direct comparability between the studies. This is the first report of pyocyanin inhibitory activity for the remaining compounds. Within the genus *Piper*, cepharadione B and benzamide from *P. pertomentellum* showed production values of 55.6% (31.2 µg/mL) and 47.9% (62.5 µg/mL), respectively [33], while 3-farnesyl-4-hydroxybenzoic acid and 2-farnesylhydroquinone from *P. bogotense* reduced production to 39% (62.5 µg/mL) and 33% (250 µg/mL) [34]. Outside the genus, inhibitory effects on pyocyanin production have also been reported for the synthetic amide N-cyclopentyl-5-(3-nitrophenyl)-5-oxo-pentamide (25% inhibition; 10 µM) [51], the sesquiterpene hydroquinone avarol (39% inhibition) [50], and vanillic acid (16% inhibition; 4 mmol/L) [52], all belonging to structural classes represented in this study.

In addition, a qualitative comparison is presented between the biofilm inhibitory activity of the isolated compounds and their corresponding source extracts, both evaluated at 62.5 μg/mL, with the caveat that direct potency comparisons between crude extracts and pure compounds are not appropriate, since the concentration units are not equivalent in biological terms. However, some general observations can be noted: *P. ceanothifolium* (inflorescences) extract was one of the few to show biofilm-inhibiting activity at 62.5 μg/mL (39.1 ± 6.3%), and several compounds isolated from this species, **C9**, **C10** and **C13,** also showed activity at the same nominal concentration (46.2 ± 7.7%, 40.6 ± 10.9% and 51.8 ± 15.1%, respectively), which is consistent with the hypothesis that bioactive constituents contribute to the observed activity of the extract, although causal attribution cannot be established from this data alone. In contrast, **C5** of *P. eriopodon* and **C7** of *P. pesaresanum* showed remarkable activity as pure compounds (53.2 ± 14.7% and 44.1 ± 7.8%, respectively), while their source extracts did not inhibit biofilm formation at the tested concentration. This result suggests that the contribution of these compounds to extract-level activity may be limited by their relative abundance within the crude matrix or modulated by interactions with other coexisting constituents [53], although these possibilities would require further investigation for confirmation.

Among the compounds evaluated, several components present in *Piper* species, including amides (**C1** and **C2**), hydroquinones (**C9**, **C10**, **C11** and **C13**) and benzoic acid derivatives (**C7** and **C8**) showed inhibitory activity in more than one of the virulence-associated phenotypes examined, suggesting a broad-spectrum modulatory profile in the concentrations analyzed. These chemical classes have previously been associated with antivirulence activity in *P. aeruginosa*, and plausible mechanisms have been proposed in the literature for some. In the case of hydroquinones, it has been reported that they can alter the integrity of the cell membrane through oxidative stress mediated by reactive oxygen species, which could be related to their inhibitory effects on biofilm formation [54]. For amides and benzoic acid derivatives, computational studies have suggested affinity for QS-associated receptor systems, supporting their potential as modulators of virulence-related signaling [55]. However, these mechanistic proposals are based on studies external to this work and cannot be directly extrapolated to the compounds evaluated here without further experimental evidence. Regarding the preliminary structure-activity trends, the comparison of **C10** and **C13,** both hydroquinones evaluated at the same concentration (31.2 μg/mL), suggests that the presence of a longer prenylate side chain in **C10** may be associated with greater inhibitory activity in multiple phenotypes. This observation is consistent with previously reported data on structurally related hydroquinones from *P. bogotense* [34] and may merit further investigation; however, given the limited number of compounds compared and the absence of data at equivalent concentrations for other pairs, a generalized structure-activity relationship cannot be established at this stage.

The results of this work demonstrate that extracts and compounds isolated from *Piper* species can reduce biofilm formation and modulate phenotypes associated with virulence, including the production of protease, elastase and pyocyanin in *P. aeruginosa* at subinhibitory concentrations. These findings are consistent with interference in QS-regulated processes, although it should be noted that the assays employed measure phenotypic outputs and do not provide direct evidence of QS inhibition at the level of signaling molecules, gene expression, or receptor binding. Mechanistic validation using transcriptomic, biochemical, or molecular coupling approaches will be necessary to establish the specific regulatory targets involved and determine whether the observed effects reflect the modulation of one or more of the interconnected QS circuits that govern virulence in this pathogen [56,57,58].

## 3. Materials and Methods

### 3.1. General Experimental Procedures

Stock solutions of the extracts and isolated compounds were prepared in dimethyl sulfoxide (DMSO; Merck, Darmstadt, Germany). The bacterial reference strains used in this study were *Pseudomonas aeruginosa* ATCC BAA-47 and *Chromobacterium violaceum* ATCC 12472, both obtained from the American Type Culture Collection (ATCC, Manassas, VA, USA). Luria–Bertani (LB) medium was used for routine growth and maintenance of the bacterial strains, whereas cetrimide medium was used as a selective medium for *P. aeruginosa*. All culture media were purchased from Liofilchem (Roseto degli Abruzzi, Italy). Round-bottom 96-well microplates (Nest, Indian Wells, CA, USA) and sterile screwcap microtubes (SureSeal™, East Brunswick, NJ, USA) were used in the bioassays. Phosphate-buffered saline (PBS) and crystal violet were purchased from Sigma-Aldrich (Burlington, MA, USA), and technical-grade ethanol (96%) was used in the biofilm quantification assay. Gentamicin (MK, Cali, Colombia) and thymol (Sigma-Aldrich, Burlington, MA, USA) were used as reference controls, depending on the assay. Microbial cultures were incubated using a temperature-controlled orbital shaker (Jeio Tech, Daejeon, Republic of Korea) and a laboratory incubator (Nuaire, Plymouth, MN, USA). Samples from the violacein inhibition assays were centrifuged using a microcentrifuge (Eppendorf, Hamburg, Germany). Optical density and absorbance measurements were recorded using a Multiskan Sky microplate spectrophotometer (Thermo Fisher Scientific, Waltham, MA, USA). Extracts were concentrated under reduced pressure using a rotary evaporator (Heidolph, Schwabach, Germany).

### 3.2. Plant Materials, Preparation of Ethanolic Extracts, and Compounds from Piper Species

#### 3.2.1. Plant Materials

Plant materials from 16 *Piper* species were collected during field trips in the departments of Cundinamarca, Boyacá, and Santander (Colombia). Commercially acquired seeds of *P. nigrum* were also included in the study. Reference specimens were sent for taxonomic identification to the Colombian National Herbarium (COL) or the Herbarium of the University of Antioquia (HUA). The collection site and taxonomic information are summarized in Table 3.

#### 3.2.2. Preparation of Ethanol Extracts

Dried and ground plant materials were extracted by maceration with 96% ethanol at room temperature. Each sample was subjected to four consecutive extraction cycles, with fresh solvent added every 4 days. After each cycle, the extract was filtered, and the solvent was removed under reduced pressure using a rotary evaporator. The resulting crude extracts were stored under refrigeration until further analysis. A total of 18 ethanolic extracts obtained from *Piper* species were included in this study (Table 3).

#### 3.2.3. Chemical Constituents from *Piper* Species

The molecules included in this study (Table 2) were previously isolated in earlier investigations of some species of the genus *Piper*. Specifically, amides **C1** to **C3** were obtained from *P. nigrum* [59]. Alkylphenols such as **C4** were isolated from *P. peltatum*, while **C5** and **C6** were obtained from *P. eriopodon* [60]. Prenylated benzoic acid derivatives such as **C7** and **C8** were isolated from *P. pesaresanum* [61]. Hydroquinones (**C9** to **C11** and **C14**) and a chromene (**C13**) were isolated from *P. ceanothifolium* [62].

### 3.3. Strains and Bacterial Growth Conditions

The reference strains were preserved at −70 °C in LB broth supplemented with 10% glycerol as a cryoprotectant. *P. aeruginosa* was cultured at 37 °C, while *C. violaceum* was cultured at 30 °C.

### 3.4. Evaluation of Extracts

#### 3.4.1. Growth Inhibition Assay in *P. aeruginosa*

The effect of the extracts on the growth of *P. aeruginosa* was evaluated using a previously reported microplate assay [33,34]. Extracts were tested at final concentrations of 1000, 250, and 62.5 µg/mL. Assays were performed in triplicate in two independent experiments. Wells containing bacterial suspension plus 2% DMSO were used as growth controls, gentamicin (2 µg/mL) was used as the inhibition control, and wells containing LB medium plus extracts and 2% DMSO without bacteria were included as sterility controls. Bacterial growth was expressed as a percentage relative to the growth control according to Equation (1).

(1) $$\%\;Growth=\left(\frac{OD\;treatment}{OD\;growth\;control}\right)\times 100$$

#### 3.4.2. Quorum Sensing Inhibition Assay in *C. violaceum*

Violacein production in *C. violaceum* was quantified using a previously reported method [33,34]. Extracts were evaluated at a final concentration of 62.5 µg/mL. Growth, medium, and inhibition controls were included, using thymol at 100 µg/mL as the positive control. All assays were performed with five replicates in two independent experiments. Violacein production was expressed as a percentage relative to the untreated control according to Equation (2).

(2) $$\%\;Violacein\;production=\left(\frac{Abs\;treatment}{Abs\;untreated\;control}\right)\times 100$$

#### 3.4.3. Biofilm Formation and Quantification Assay in *P. aeruginosa*

Biofilm formation by *P. aeruginosa* was quantified using the crystal violet staining method, as previously reported [33,34]. Extracts were evaluated at a final concentration of 62.5 µg/mL. Quercetin (3.9 µg/mL) was used as the positive control, and 2% DMSO was used as the vehicle control. Additional controls included untreated bacterial cultures and medium blanks. All assays were performed with five replicates in two independent experiments. Absorbance was measured at 570 nm, and biofilm formation was expressed as a percentage relative to the untreated control according to Equation (3).

(3) $$\%\;Biofilm\;formation=\left(\frac{Abs\;treatment}{Abs\;untreated\;control}\right)\times 100$$

### 3.5. Evaluation of Compounds

#### 3.5.1. Growth Inhibition Assay in *P. aeruginosa*

The effect of compounds on the growth of *P. aeruginosa* was evaluated as described in Section 3.4.1, with the following modification: compounds were tested at final concentrations ranging from 250 to 15.6 µg/mL.

#### 3.5.2. Biofilm Formation and Quantification Assay in *P. aeruginosa*

The effect of compounds on biofilm formation by *P. aeruginosa* was evaluated as described in Section 3.4.3, with the following modification: compounds were tested at final concentrations ranging from 250 to 15.6 µg/mL.

#### 3.5.3. Evaluation of Virulence Factor Production in *P. aeruginosa*

The effects of *Piper*-derived compounds on the production of virulence factors by *P. aeruginosa* were evaluated following previously reported methodologies [36,43]. Compounds were tested at concentrations ranging from 250 to 62.5 µg/mL for 24 h. Cinnamic acid at 250 µg/mL was used as a positive control [49]. All assays were performed in five replicates, and the results were expressed as percentages relative to the untreated control.

##### Determination of the Pyocyanin Production

Pyocyanin production was determined according to a previously reported methodology [63]. Briefly, 750 µL of culture supernatant was extracted with 375 µL of chloroform. The organic phase was then acidified with 300 µL of 0.2 M HCl, resulting in a pink aqueous phase. An aliquot of 150 µL of this phase was mixed with 150 µL of 200 mM Tris buffer, and absorbance was measured at 390 nm in a 96-well microplate.

##### Determination of the Elastase Production

Elastase production was quantified as previously described [63]. Briefly, 25 µL of culture supernatant was mixed with 225 µL of elastin–Congo red suspended in 100 mM Tris buffer (pH 7.5) and incubated for 3 h at 37 °C under agitation. Subsequently, PBS buffer (pH 6.0) was added to stop the reaction, and the mixture was allowed to cool for 2 min. Samples were then centrifuged at 10,000 rpm for 10 min to remove the insoluble substrate, and the absorbance of the supernatant was measured at 495 nm.

##### Determination of the Protease Production

Protease production was quantified following a previously described method [63]. Briefly, 37.5 µL of culture supernatant was mixed with 100 mM Tris buffer (pH 8.0) containing 0.3% azocasein and incubated statically for 1 h at 37 °C. The reaction was stopped by adding 10% trichloroacetic acid (TCA). The mixture was then centrifuged at 10,000 rpm for 10 min, and the absorbance of the supernatant was measured at 490 nm.

### 3.6. Statistical Analysis

Data were analyzed by one-way analysis of variance (ANOVA), after assessing the assumptions of normality (Shapiro–Wilk test), homogeneity of variances (Levene’s test), and the presence of outliers (Chauvenet criterion); any data point identified as an outlier was excluded prior to further analysis. When all assumptions were met, one-way ANOVA followed by Tukey’s Honestly Significant Difference (HSD) post hoc test was applied to identify differences among groups relative to untreated bacterial control. When the assumptions of normality or homogeneity of variances were not met, the Kruskal–Wallis test followed by Dunn’s post hoc correction was applied as a non-parametric alternative. Data are presented as mean ± standard deviation. Statistical analyses were performed using RStudio 2024.04.2, and differences were considered statistically significant at *p* < 0.05.

## 4. Conclusions

The present research contributes to the characterization of the anti-QS potential of extracts from *Piper* species, establishing that extracts of *P. aduncum*, *P. cumanense*, *P. grande*, and *P. sucrense* significantly inhibited both violacein production in *C. violaceum* and biofilm formation in *P. aeruginosa*, while *P. ceanothifolium* extract significantly inhibited only biofilm formation. Hydroquinone-type compounds and benzoic acid derivatives had inhibitory effects on the production of virulence factors and biofilm formation in *P. aeruginosa*.

Future studies should focus on mechanistic validation through transcriptomic or molecular docking approaches to identify specific QS regulatory targets. Additionally, in vivo models and cytotoxicity evaluations of the most active compounds will be necessary to assess their therapeutic potential. These findings contribute to the growing body of evidence supporting *Piper* species as a promising botanical resource for the development of anti-virulence strategies against drug-resistant *P. aeruginosa*.

## Figures and Tables

**Table 1:** Inhibitory effect of ethanolic extracts of *Piper* on *P. aeruginosa* biofilm formation and violacein production by *C. violaceum*.

Extracts	% Biofilm Formation on *P. aeruginosa*	% Violacein Production in *C. violaceum*
	Concentration Tested 62.5 µg/mL	
*P. annulatispicum* (L)	71.8 ± 6.9	62.6 ± 6.9 *
*P. aduncum* (L)	49.2 ± 6.1 *	45.7 ± 2.8 *
*P. aduncum* (I)	45.9 ± 9.1 *	92.9 ± 6.7
*P. aduncum* (ST)	39.2 ± 8.7 *	89.5 ± 7.8
*P. ceanothifolium* (I)	39.1 ± 6.3 *	79.3 ± 4.2
*P. cumanense* (AP)	70.8 ± 2.8 *	54.9 ± 4.1 *
*P. cundinamarcanum* (AP)	69.4 ± 13.9	54.1 ± 3.7 *
*P. eriopodon* (AP)	92.1 ± 9.8	93.3 ± 14.5
*P. falcifolium* (L)	87.1 ± 11.1	66.1 ± 7.5 *
*P. grande* (L)	65.1 ± 9.4 *	45.1 ± 4.8 *
*P. haughtii* (L)	68.9 ± 3.3	49.9 ± 2.3 *
*P. nigrum* (SE)	100.0 ± 17.1	31.5 ± 4.8 *
*P. peltatum* (I)	100.0 ± 16.3	76.1 ± 9.3 *
*P. pertomentellum* (ST)	9.1 ± 8.6	71.4 ± 3.9 *
*P. pesaresanum* (AP)	100.0 ± 6.5	44.4 ± 5.9 *
*P. statarium* (AP)	87.9 ± 9.5	75.5 ± 5.1 *
*P. sucrense* (L)	55.7 ± 5.1 *	61.5 ± 2.7 *
*P. umbellatum* (L)	76.9 ± 11.7 *	74.6 ± 4.2 *
Quercetin (3.9 µg/mL)	29.9 ± 10.4	-
Thymol (100 µg/mL)	-	0 ± 0.0

L: leaves, ST: stems, AP: aerial part, I: inflorescences, NI: Not inhibited. * Statistically significant reduction relative to the untreated control (Tukey’s HSD test, *p* < 0.05).

**Table 2:** Effect of compounds isolated from *Piper* species on biofilm formation and virulence factor production of *P. aeruginosa*.

Code	Compound	Species	Structure	Concentration Tested	% Formation	% Production		
				µg/mL/µM	Biofilm	Protease	Elastase	Pyocyanin
**C1**	Piperine	*P. nigrum*	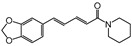	**125/438.5**	61.5 ± 12.9	57.2 ± 2.6	43.3 ± 9.6	27.6 ± 5.3
**C2**	Piperlonguminine		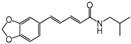	**250/915.7**	51.4 ± 14.7	64.2 ± 10.8	43.8 ± 7.4	35.2 ± 9.2
**C3**	Isopiperine		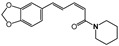	**125/438**	113.1 ± 1	74.5 ± 19.2	55.7 ± 21.1	69.5 ± 48.2
**C4**	4-Nerolidylcathecol	*P. peltatum*	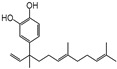	**62.5/199.1**	142.2 ± 1	97.4 ± 1	65.4 ± 8.9	61.6 ± 13.5
**C5**	Eriopodol A	*P. eriopodon*	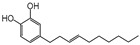	**62.5/252**	53.2 ± 14.7	99.3 ± 3.1	141.3 ± 21.4	134.6 ± 28.9
**C6**	Gibbilimbol B		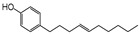	**15.6/67.2**	28.1 ± 2.6	92.2 ± 5.2	51.2 ± 7.9	22.7 ± 4.5
**C7**	4-Methoxynervogenic acid	*P. pesaresanum*	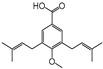	**31.2/108.3**	38.6 ± 10.2	84.1 ± 19.9	58.2 ± 16.1	53.3 ± 15.8
**C8**	3-(3′,3′-dimethylallyl-1′-oxo)-5-(3″,3″-dimethylallyl)-4-hydroxybenzoic acid		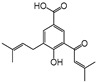	**250/868.1**	50.6 ± 9.6	57.3 ± 17.1	67.5 ± 42.4	49.2 ± 32.9
**C9**	1,4-dihydroxy-2-(3′-hydroxy-3′7′-dimethyl-1′-oxo-6′-octenyl)benzene	*P. ceanothifolium*	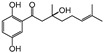	**31.2/112.2**	53.1 ± 16.5	79.4 ± 17.9	64.4 ± 14.2	64.4 ± 19.1
**C10**	Debromocymopolone		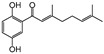	**31.2/120**	43.9 ± 4.1	49.7 ± 10.6	25.9 ± 14.4	32.6 ± 2.5
**C11**	1,4-dihydroxy-2-(3′,7′-dimethyl-1′-oxo-2′-Z-6′-octadienyl)benzene		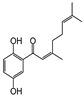	**125/480.7**	44.2 ± 5.3	68.7 ± 25.8	40.2 ± 2.5	44.3 ± 12.4
**C12**	Lhotzchromene		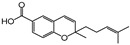	**250/919.1**	102.3 ± 29.8	75.2 ± 21.3	73.1 ± 19.5	63.1 ± 30.6
**C13**	1,4-dihydroxy-2-(1′-hydroxy-1′-methyl-1′-ethyl)benzene		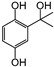	**31.2/185.7**	44.5 ± 10.1	79.3 ± 13.0	47.2 ± 12.5	58.8 ± 16.7
**Positive Controls**	Cinnamic acid		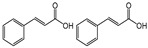	**250/1689**	NT	60.2 ± 14.4	42.6 ± 6.4	32.3 ± 5.2
	Quercetin		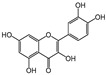	**3.9/12.9**	29.9 ± 17.4	NT	NT	NT

NT: Not tested.

**Table 3:** Taxonomic identity and botanical origin of extracts from Colombian *Piper* species evaluated for their inhibitory potential on quorum sensing and biofilm formation.

Species	Organ	Place of Origin	Herbarium Code
*Piper annulatispicum* Trel. & Yunck. **	Leaves	Otanche (Boyacá)	HUA 217579
*Piper aduncum* L. **	Leaves	San Mateo (Boyacá)	COL579924
	Inflorescences		
	Stems		
*Piper ceanothifolium* Kunt. **	Inflorescences	El Colegio (Cundinamarca)	HUA 217583
*Piper cumanense* Kunt. **	Aerial part	Arcabuco (Boyacá)	HUA217578
*Piper cundinamarcanum* Trel. **	Leaves	Apulo (Cundinamarca)	HUA217592
*Piper eriopodon* Miq. **	Aerial part	Arcabuco (Boyacá)	Identified without Voucher
*Piper falcifolium* Trel. *	Leaves	Otanche (Boyacá)	HUA 217600
*Piper grande* Vahl. **	Leaves	Otanche (Boyacá)	HUA 217596
*Piper haughtii* Trel. & Yunck. **	Leaves	Otanche (Boyacá)	HUA 217598
*Piper nigrum* L.	Seed	Pimienta Pepper trading S.A.S.	NA
*Piper peltatum* L. *	Inflorescences	Viotá (Cundinamarca)	Identified without Voucher
*Piper pertomentellum* T. & Y. *	Stems	San Mateo (Boyacá)	Identified without Voucher
*Piper pesaresanum* C.DC. **	Aerial part	Santa Bárbata (Santander)	COL553307
*Piper statarium* Trel. & Yunck. **	Aerial part	Otanche (Boyacá)	HUA 217602
*Piper sucreense* Trel. & Yunck. **	Leaves	Otanche (Boyacá)	HUA 217595
*Piper umbellatum* L. *	Leaves	Viotá (Cundinamarca)	Identified without Voucher

* Genetic resource access contract (CARG), ** Amnesty (Article 6 of Law 1955 of 2019), NA: not applicable.

## Data Availability

The original contributions presented in this study are included in the article and Supplementary Materials. Further inquiries can be directed to the corresponding author.

## Supplementary Materials

The following supporting information can be downloaded at: https://www.mdpi.com/article/10.3390/antibiotics15060627/s1, Table S1: Percentage growth of *P. aeruginosa* in the presence of extract of *Piper* species; Table S2: Percentage growth of *P. aeruginosa* in response to the evaluation of compounds derived from *Piper* species.

## References

[1] Yang J., Xu J.-F., Liang S. (2025). Antibiotic resistance in *Pseudomonas aeruginosa*: Mechanisms and emerging treatment. Crit. Rev. Microbiol..

[2] Touati A., Ibrahim N.A., Tighilt L., Idres T. (2025). Anti-QS Strategies Against *Pseudomonas aeruginosa* Infections. Microorganisms.

[3] Maiga A., Ampomah-Wireko M., Li H., Fan Z., Lin Z., Zhen H., Kpekura S., Wu C. (2025). Multidrug-resistant bacteria quorum-sensing inhibitors: A particular focus on *Pseudomonas aeruginosa*. Eur. J. Med. Chem..

[4] Ramatla T., Nkhebenyane J., Lekota K.E., Thekisoe O., Monyama M., Achilonu C.C., Khasapane G. (2025). Global prevalence and antibiotic resistance profiles of carbapenem-resistant *Pseudomonas aeruginosa* reported from 2014 to 2024: A systematic review and meta-analysis. Front. Microbiol..

[5] Oliver A., Arca-Suárez J., Gomis-Font M.A., González-Pinto L., López-Causapé C. (2025). Emerging resistance mechanisms to newer β-lactams in *Pseudomonas aeruginosa*. Clin. Microbiol. Infect..

[6] Mohommed Saleh F., Abbas Mohammed H. (2025). Variations in *Pseudomonas aeruginosa* Biofilm Formation Influence Virulence and amoxicillin Resistance. World J. Exp. Biosci..

[7] Giovagnorio F., De Vito A., Madeddu G., Parisi S.G., Geremia N. (2023). Resistance in *Pseudomonas aeruginosa*: A Narrative Review of Antibiogram Interpretation and Emerging Treatments. Antibiotics.

[8] Qin S., Xiao W., Zhou C., Pu Q., Deng X., Lan L., Liang H., Song X., Wu M. (2022). *Pseudomonas aeruginosa*: Pathogenesis, virulence factors, antibiotic resistance, interaction with host, technology advances and emerging therapeutics. Signal Transduct. Target. Ther..

[9] Zhang X., Zhang D., Zhou D., Zheng S., Li S., Hou Q., Li G., Han H. (2025). A comprehensive review of the pathogenic mechanisms of Pseudomonas aeruginosa: Synergistic effects of virulence factors, quorum sensing, and biofilm formation. Front. Microbiol..

[10] Pang Z., Raudonis R., Glick B.R., Lin T.J., Cheng Z. (2019). Antibiotic resistance in *Pseudomonas aeruginosa*: Mechanisms and alternative therapeutic strategies. Biotechnol. Adv..

[11] Zhao X., Yu Z., Ding T. (2020). Quorum-Sensing Regulation of Antimicrobial Resistance in Bacteria. Microorganisms.

[12] Bhuiyan M.N.I. (2025). Biofilm, resistance, and quorum sensing: The triple threat in bacterial pathogenesis. Microbe.

[13] Papenfort K., Bassler B.L. (2016). Quorum-Sensing Signal-Response Systems in Gram-Negative Bacteria. Nat. Rev. Microbiol..

[14] Bassler B.L., Losick R. (2006). Bacterially speaking. Cell.

[15] Letizia M., Diggle S.P., Whiteley M. (2025). *Pseudomonas aeruginosa*: Ecology, evolution, pathogenesis and antimicrobial susceptibility. Nat. Rev. Microbiol..

[16] Wang Y., Xiao Q., Yang Q., Long Y., Jiang Z., Zhang T., Hu Y., Gao B., Chen X., Wang T. (2025). Impact of Pseudomonas aeruginosa biofilm formation by different sequence types on treating lower limb vascular infections. Curr. Res. Microb. Sci..

[17] Vadakkan K., Sathishkumar K., Mapranathukaran V.O., Ngangbam A.K., Nongmaithem B.D., Hemapriya J., Nair J.B. (2024). Critical review on plant-derived quorum sensing signaling inhibitors in *Pseudomonas aeruginosa*. Bioorg. Chem..

[18] Carette J., Nachtergael A., Duez P., El Jaziri M., Rasamiravaka T. (2020). Natural Compounds Inhibiting *Pseudomonas aeruginosa* Biofilm Formation by Targeting Quorum Sensing Circuitry. Bacterial Biofilms.

[19] Shariati A., Noei M., Askarinia M., Khoshbayan A., Farahani A., Chegini Z. (2024). Inhibitory effect of natural compounds on quorum sensing system in *Pseudomonas aeruginosa*: A helpful promise for managing biofilm community. Front. Pharmacol..

[20] Parmar V.S., Jain S.C., Bisht K.S., Jain R., Taneja P., Jha A., Tyagi O.D., Prasad A.K., Wengel J., Olsen C.E. (1997). Phytochemistry of the genus *Piper*. Phytochemistry.

[21] Perez Gutierrez M.R., Neira Gonzalez M.A., Hoyo-Vadillo C. (2013). Alkaloids from *Piper*: A Review of its Phytochemistry and Pharmacology. Mini Rev. Med. Chem..

[22] Salehi B., Zakaria Z.A., Gyawali R., Ibrahim S.A., Rajkovic J., Shinwari Z.K., Khan T., Sharifi-Rad J., Ozleyen A., Turkdonmez E. (2019). Piper Species: A Comprehensive Review on Their Phytochemistry, Biological Activities and Applications. Molecules.

[23] Navickiene H.M.D., Morandim A.d.A., Alécio A.C., Regasini L.O., Bergamo D.C.B., Telascrea M., Cavalheiro A.J., Lopes M.N., Bolzani V.d.S., Furlan M. (2006). Composition and antifungal activity of essential oils from *Piper aduncum*, *Piper arboreum* and *Piper tuberculatum*. Quim. Nova.

[24] Durant-Archibold A.A., Santana A.I., Gupta M.P. (2018). Ethnomedical uses and pharmacological activities of most prevalent species of genus Piper in Panama: A review. J. Ethnopharmacol..

[25] Olivero V.J.T., Pájaro C.N.P., Stashenko E. (2011). Antiquorum sensing activity of essential oils isolated from different species of the genus *Piper*. Vitae.

[26] Tan L.Y., Yin W.F., Chan K.G. (2013). *Piper nigrum*, *Piper betle* and *Gnetum gnemon*- Natural food sources with anti-quorum sensing properties. Sensors.

[27] Datta S., Jana D., Maity T.R., Samanta A., Banerjee R. (2016). *Piper betle* leaf extract affects the quorum sensing and hence virulence of *Pseudomonas aeruginosa* PAO1. 3 Biotech.

[28] Sikdar B., Mukherjee S., Bhattacharya R., Raj A., Roy A., Banerjee D., Gangopadhyay G., Roy S. (2024). The anti-quorum sensing and biofilm inhibitory potential of *Piper betle* L. leaf extract and prediction of the roles of the potent phytocompounds. Microb. Pathog..

[29] Schlichter Kadosh Y., Muthuraman S., Nisaa K., Ben-Zvi A., Karsagi Byron D.L., Shagan M., Brandis A., Mehlman T., Gopas J., Saravana Kumar R. (2024). *Pseudomonas aeruginosa* quorum sensing and biofilm attenuation by a di-hydroxy derivative of piperlongumine (PL-18). Biofilm.

[30] Rangel-Ch J.O. (2015). La Riqueza De Las Plantas Con Flores De Colombia: The richness of flowering plants in Colombia. Caldasia.

[31] Trujillo W., Vargas V., Parra C. (2022). Las Especies de Piper: En la Vertiente Amazónica de los Andes, Caquetá. Guía de Campo.

[32] Bernal R., Gradstein S.R., Celis M. (2015). Catálogo de Plantas y Líquenes de Colombia.

[33] Hernández-Moreno L.V., Pabón-Baquero L.C., Prieto-Rodriguez J.A., Patiño-Ladino O.J. (2023). Bioactive Compounds from *P. pertomentellum* That Regulate QS, Biofilm Formation and Virulence Factor Production of *P. aeruginosa*. Molecules.

[34] Sierra-Quitian A.G., Hernandez-Moreno L.V., Pabon-Baquero L.C., Prieto-Rodriguez J.A., Patiño-Ladino O.J. (2023). Antiquorum and Antibiofilm Activities of *Piper bogotense* C. DC. against *Pseudomonas aeruginosa* and Identification of Bioactive Compounds. Plants.

[35] El-Mowafy S.A., Abd El Galil K.H., Habib E.S.E., Shaaban M.I. (2017). Quorum sensing inhibitory activity of sub-inhibitory concentrations of β-lactams. Afr. Health Sci..

[36] Aleanizy F.S., Alqahtani F.Y., Eltayb E.K., Alrumikan N., Almebki R., Alhossan A., Almangour T.A., AlQahtani H. (2021). Evaluating the effect of antibiotics sub-inhibitory dose on *Pseudomonas aeruginosa* quorum sensing dependent virulence and its phenotypes. Saudi J. Biol. Sci..

[37] Bahari S., Zeighami H., Mirshahabi H., Roudashti S., Haghi F. (2017). Inhibition of *Pseudomonas aeruginosa* quorum sensing by subinhibitory concentrations of curcumin with gentamicin and azithromycin. J. Glob. Antimicrob. Resist..

[38] Rather M.A., Saha D., Bhuyan S., Jha A.N., Mandal M. (2022). Quorum Quenching: A Drug Discovery Approach Against *Pseudomonas aeruginosa*. Microbiol. Res..

[39] Vázquez-Martínez J., Buitemea-Cantúa G.V., Gutierrez-Villagomez J.M., García-González J.P., Ramírez-Chávez E., Molina-Torres J. (2020). Bioautography and GC-MS based identification of piperine and trichostachine as the active quorum quenching compounds in black pepper. Heliyon.

[40] Vallejo A., Feitosa A., Gourlart A., Pires L., Mosquera O. (2014). Tamizaje de acción antimicrobiana de 34 extractos vegetales contra bacilos gramnegativos. Salud Soc. Uptc.

[41] Kloucek P., Polesny Z., Svobodova B., Vlkova E., Kokoska L. (2005). Antibacterial screening of some Peruvian medicinal plants used in Callería District. J. Ethnopharmacol..

[42] Purayil S.K., Annley C., Ponnaiah P., Pattammadath S., Javad P.T.M., Jenifer Selvarani A., Raji P., Thirumurugan R., Iyappan P., Samrot A.V. (2019). Evaluation of antioxidant and antimicrobial activity of some plants collected from Malaysia. J. Pure Appl. Microbiol..

[43] Ahmed R.H. (2024). Emerging Patterns of Meropenem Resistance in *Pseudomonas aeruginosa* from Burn Patients: Epidemiological Data from Central Iraq. World J. Exp. Biosci..

[44] Mulat M., Banicod R.J.S., Tabassum N., Javaid A., Karthikeyan A., Jeong G.-J., Kim Y.-M., Jung W.-K., Khan F. (2025). Multiple Strategies for the Application of Medicinal Plant-Derived Bioactive Compounds in Controlling Microbial Biofilm and Virulence Properties. Antibiotics.

[45] Dzięgielewska M., Tomczyk M., Wiater A., Woytoń A., Junka A. (2025). Targeting Ocular Biofilms with Plant-Derived Antimicrobials in the Era of Antibiotic Resistance. Molecules.

[46] Chadha J., Harjai K., Chhibber S. (2022). Revisiting the virulence hallmarks of Pseudomonas aeruginosa: A chronicle through the perspective of quorum sensing. Environ. Microbiol..

[47] Rybtke M., Hultqvist L.D., Givskov M., Tolker-Nielsen T. (2015). *Pseudomonas aeruginosa* Biofilm Infections: Community Structure, Antimicrobial Tolerance and Immune Response. J. Mol. Biol..

[48] Skariyachan S., Sridhar V.S., Packirisamy S., Kumargowda S.T., Challapilli S.B. (2018). Recent perspectives on the molecular basis of biofilm formation by *Pseudomonas aeruginosa* and approaches for treatment and biofilm dispersal. Folia Microbiol..

[49] Rajkumari J., Borkotoky S., Murali A., Suchiang K., Mohanty S.K., Busi S. (2018). Cinnamic acid attenuates quorum sensing associated virulence factors and biofilm formation in Pseudomonas aeruginosa PAO1. Biotechnol. Lett..

[50] Pejin B., Iodice C., Tommonaro G., Stanimirovic B., Ciric A., Glamoclija J., Nikolic M., Rosa S., Sokovic M. (2014). Further in vitro Evaluation of Antimicrobial Activity of the Marine Sesquiterpene Hydroquinone Avarol. Curr. Pharm. Biotechnol..

[51] Hossain M.A., Sattenapally N., Parikh H.I., Li W., Rumbaugh K.P., German N.A. (2020). Design, synthesis, and evaluation of compounds capable of reducing Pseudomonas aeruginosa virulence. Eur. J. Med. Chem..

[52] Ugurlu A., Karahasan Yagci A., Ulusoy S., Aksu B., Bosgelmez-Tinaz G. (2016). Phenolic compounds affect production of pyocyanin, swarming motility and biofilm formation of *Pseudomonas aeruginosa*. Asian Pac. J. Trop. Biomed..

[53] Castillo-Juárez I., García-Contreras R., Velázquez-Guadarrama N., Soto-Hernández M., Martínez-Vázquez M. (2013). Amphypterygium adstringens Anacardic Acid Mixture Inhibits Quorum Sensing-controlled Virulence Factors of *Chromobacterium violaceum* and *Pseudomonas aeruginosa*. Arch. Med. Res..

[54] Jeyanthi V., Velusamy P., Kumar G.V., Kiruba K. (2021). Effect of naturally isolated hydroquinone in disturbing the cell membrane integrity of *Pseudomonas aeruginosa* MTCC 741 and *Staphylococcus aureus* MTCC 740. Heliyon.

[55] Victoria-Munoz F., Sanchez-Cruz N., Medina-Franco J.L., Lopez-Vallejo F. (2022). Cheminformatics analysis of molecular datasets of transcription factors associated with quorum sensing in *Pseudomonas aeruginosa*. RSC Adv..

[56] Bassetti S., Tschudin-Sutter S., Egli A., Osthoff M. (2022). Optimizing antibiotic therapies to reduce the risk of bacterial resistance. Eur. J. Intern. Med..

[57] Aljeldah M.M. (2022). Antimicrobial Resistance and Its Spread Is a Global Threat. Antibiotics.

[58] Bramhachari P.V., Bramhachari P.V. (2019). Implication of Quorum Sensing and Biofilm Formation in Medicine, Agriculture and Food Industry.

[59] Rodríguez-Rodríguez D.E. (2026). Búsqueda de Agentes Fitosanitarios Para El Control de *Sitophilus Zeamais* Basado En Las Amidas Provenientes de *Piper Nigrum* (Piperaceae). Master’s Thesis.

[60] Ladino-Vargas C. (2026). Búsqueda de Agentes Fitosanitarios Provenientes Del Género *Piper* Para El Control de Microorganismos Que Afectan El Cultivo de Cacao (*Theobroma Cacao*) L.. Ph.D. Thesis.

[61] Chitiva-Chitiva L.C., Ladino-Vargas C., Cuca-Suárez L.E., Prieto-Rodríguez J.A., Patiño-Ladino O.J. (2021). Antifungal Activity of Chemical Constituents from Piper pesaresanum C. DC. and Derivatives against Phytopathogen Fungi of Cocoa. Molecules.

[62] Mahecha-Jimenez Y.S., Patiño-Ladino O.J., Prieto-Rodríguez J.A. (2025). Chemical Constituents and Antifungal Properties of Piper ceanothifolium Kunth Against Phytopathogens Associated with Cocoa Crops. Plants.

[63] Filloux A., Ramos J., Filloux A., Ramos J.-L. (2014). Pseudomonas Methods and Protocols Methods in Molecular Biology.

