# Supplementary material for: Inhibition of Quorum Sensing-Controlled Virulence Factors and Biofilm in Pseudomonas aeruginosa by Piper Species
Source: Antibiotics (Basel). 2026 Jun 22;15(6):627. doi: 10.3390/antibiotics15060627 (PMC13295477; doi:10.3390/antibiotics15060627)
Supplement: Supplementary file 1 [file antibiotics-15-00627-s001.zip › antibiotics-4306182-supplementary.pdf]

**Supplementary material**

# Inhibition of Quorum Sensing-Controlled Virulence Factors and Biofilm in *Pseudomonas aeruginosa* by *Piper* Species

**Juliet A. Prieto-Rodriguez[1,*], Lida V. Hernández-Moreno[2], Ludy C. Pabón-Baquero[3], Oscar J. Patiño-Ladino[2], Luis E. Cuca-Suárez[2]**

[1] Departamento de Química, Facultad de Ciencias, Pontificia Universidad Javeriana, Bogotá 110231, Colombia
[2] Departamento de Química, Facultad de Ciencias, Universidad Nacional de Colombia, Sede Bogotá, Bogotá 111321, Colombia; lhernandezmo@unal.edu.co (L.V.H-M.), ojpatinol@unal.edu.co (O.J.P-L.), lecucas@unal.educ.co (L.E.C-S.)
[3] Escuela de Ciencias Básicas y Aplicadas, Universidad de la Salle, Bogotá 111711, Colombia; lupabon@unisalle.edu.co (L.C.P-B.)
* Correspondence: juliet.prieto@javeriana.edu.co (J.A.P-R), Tel.: +57-6013208320 (ext. 4124)

## Table of contents

**1.** Growth of *P. aeruginosa* in the presence of extract of *Piper* species.
**2.** Growth of *P. aeruginosa* in the presence of compounds of *Piper* species.

**Table S1**. Percentage growth of *P. aeruginosa* in the presence of extract of *Piper* species.

| Code | Extracts | Concentrations (ppm) and % growth results | | |
|---|---|---|---|---|
| | | **1000** | **250** | **62.5** |
| **E1** | *P. annulatispicum* (L) | 100.5 ± 0.9 | 112.1 ± 2.1 | 109.2± 1.1 |
| **E2** | *P. aduncum* (L) | 108.7 ± 1.7 | 109.4 ± 2.2 | 117.1 ± 1.1 |
| **E3** | *P. aduncum* (I) | 73.4 ± 6.1 * | 87.8 ± 4.1 | 105.2 ± 12.5 |
| **E4** | *P. aduncum* (ST) | 87.5 ± 9.3 | 97.9 ± 12.5 | 116.1 ± 6.1 |
| **E5** | *P. ceanothifolium* (I) | 72.5 ± 1.3* | 87.2 ± 4.9 * | 109.5 ± 5.5 |
| **E6** | *P. cumanense* (AP) | 32.6 ± 11.1* | 82.8 ± 13.6 * | 97.9 ± 4.1 |
| **E7** | *P. cundinamarcanum* (AP) | 103.3 ± 2.1 | 105.3 ± 0.3 | 100.3 ± 3.1 |
| **E8** | *P. eriopodon* (AP) | 87.5 ± 0.5* | 89.1 ± 3.1 | 101.7 ± 0.7 |
| **E9** | *P. falcifolium* (L) | 83.8 ± 3.4* | 108.3 ± 1.5 | 109.3 ± 1.1 |
| **E10** | *P. grande* (L) | 104.9 ± 2.5 | 106.6 ± 2.1 | 100.1 ± 12.7 |
| **E11** | *P. haughtii* (L) | 102.4 ± 1.55 | 109.5 ± 0.9 | 106.6 ± 0.13 |
| **E12** | *P. nigrum* (SE) | 75.5 ± 0.2* | 88.8 ± 9.6 | 110.4 ± 11.1 |
| **E13** | *P. peltatum* (I) | 51.8 ± 5.9 * | 69.4 ± 5.5* | 90.9 ± 1.3 |
| **E14** | *P. pertomentellum* (ST) | 129.3 ± 9.8 | 132.2 ± 1.3 | 122.1 ± 6.6 |
| **E15** | *P. pesaresanum* (AP) | 100.6 ± 2.5 | 108.2 ± 2.9 | 111.6 ± 0.9 |
| **E16** | *P. statarium* (AP) | 70.3 ± 6.1* | 94.1 ± 0.2 | 100.8 ± 0.5 |
| **E17** | *P. sucrense* (L) | 97.5 ± 3.2 | 91.4 ± 4.8 | 100.4 ± 5.5 |
| **E18** | *P. umbelatum* (L) | 3.5 ± 10.3* | 68.1± 19.2 * | 97.1 ± 14.6 |
| **Growth control** | Bacteria | 100.1 ± 0.1 | | |
| **Positive Control** | Gentamicine (2 ppm) | 2.3 ± 0.2 | | |

Data are represented the mean ± standard deviation of five independent replicates.

.

**Table S2.** Percentage growth of *P. aeruginosa* in response to the evaluation of compounds derived from *Piper* species.

| Code | Compound | Species | Concentrations (ppm) | % growth |
|---|---|---|---|---|
| **C1** | Piperine | *P. nigrum* | 250 | 89.4 ± 6.2 |
| | | | 125 | 78.2 ± 3.2 |
| | | | 62.5 | 78.8 ± 3.8 |
| **C2** | Piperlonguminine | | 250 | 114.5 ± 8.5 |
| | | | 125 | 105.7 ± 8.4 |
| | | | 62.5 | 95.5 ± 7.3 |
| **C3** | Isopiperine | | 250 | 104.8 ± 2.3 |
| | | | 125 | 76.7 ± 3.5 |
| | | | 62.5 | 65.1 ± 2.9 |
| **C4** | 4-Nerolidylcathecol | *P. peltatum* | 250 | 103.9 ± 5.2 |
| | | | 125 | 64.4 ± 8.6 |
| | | | 62.5 | 60.7 ± 5.2 |
| **C5** | Eriopodol A | *P. eriopodon* | 250 | 99.5 ± 9.4 |
| | | | 125 | 72.7 ± 3.5 |
| | | | 62.5 | 74.4 ± 10.1 |
| **C6** | Gibbilimbol B | | 250 | 86.1 ± 7.2 |
| | | | 125 | 83.9 ± 6.6 |
| | | | 62.5 | 87.3 ± 1.5 |
| **C7** | 4-Methoxynervogenic acid | *P. pesaresanum* | 250 | 99.7 ± 2.6 |
| | | | 125 | 75.6 ± 4.2 |
| | | | 62.5 | 67.9 ± 6.4 |
| **C8** | 3-(3'.3'-dimethylallyl-1'-oxo)-5-(3''.3''-dimethylallyl)-4-hydroxybenzoic acid | | 250 | 76.2 ± 9.6 |
| | | | 125 | 63.2 ± 9.2 |
| | | | 62.5 | 79.7 ± 4.5 |
| **C9** | 1.4-dihydroxy-2- (3'-hydroxy-3' 7'-dimethyl-1'-oxo-6'-octenyl)benzene | *P. ceanothifolium* | 250 | 39.6 ± 5.4 |
| | | | 125 | 64.2 ± 6.2 |
| | | | 62.5 | 61.1 ± 8.9 |
| **C10** | Debromocymopolone | | 250 | 106.7 ± 8.4 |
| | | | 125 | 70.8 ± 17.2 |
| | | | 62.5 | 62.9 ± 13.9 |
| **C11** | 1.4-dihydroxy-2- (3 '. 7'-dimethyl-1'-oxo-2'-Z-6'-octadienyl)benzene | | 250 | 90.1 ± 9.4 |
| | | | 125 | 69.4 ± 8.7 |
| | | | 62.5 | 73.2 ± 5.7 |

| | | | | |
|---|---|---|---|---|
| **C12** | Lhotzchromene | | 250 | 96.8 ± 7.9 |
| | | | 125 | 99.4 ± 10.9 |
| | | | 62.5 | 103.1 ± 6.4 |
| **C13** | 1.4-dihydroxy-2-(1'-hydroxy-1'-methyl-1'-ethyl)benzene | | 250 | 98.7 ± 5.5 |
| | | | 125 | 110.8 ± 10.1 |
| | | | 62.5 | 106.1 ± 11.2 |
| **Growth control** | Bacterial | | - | 100.1 ± 3.2 |
| **Positive Control** | Quercetine | | 3.9 | 99.1 ± 2.7 |

Data are represented the mean ± standard deviation of five independent replicates.
